# Resetting of quartz and feldspar luminescence signals under water

**DOI:** 10.1038/s41598-026-44245-6

**Published:** 2026-03-17

**Authors:** Anna-Maartje de Boer, Natascia Pannozzo, Stuart G. Pearson, Tjitske J. Kooistra, Bram van Prooijen, Jakob Wallinga

**Affiliations:** 1 Soil Geography and Landscape Group and Netherlands Centre for Luminescence Dating, Wageningen University and Research, Droevendaalsesteeg 3, 6708 PB Wageningen, The Netherlands; 2 Faculty of Civil Engineering and Geosciences, Delft University of Technology, Stevinweg 1, 2628 CN Delft, The Netherlands; 3 Department of Estuarine and Delta Systems, Royal Netherlands Institute for Sea Research, Korringaweg 7, 4401 NT Yerseke, The Netherlands

**Keywords:** Environmental sciences, Ocean sciences, Solid Earth sciences

## Abstract

Quantifying luminescence signal resetting of sand grains in turbid waters is essential for both sediment dating and tracing, yet direct measurement under natural subaqueous conditions remain scarce. Here, we present the first depth-resolved experiment that combines in-situ luminescence resetting, subaqueous light spectra and suspended sediment concentration in a tidal inlet. Sand-sized quartz and feldspar grains were exposed to daylight at multiple depths during a one-day deployment, while optical and sediment conditions were continuously monitored. Single-grain luminescence measurements reveal depth-dependent resetting with a bleaching front below which no significant signal resetting occurs within a day. The position of this bleaching front depends on signal bleachability and agrees with predictions based on spectral irradiance and mineral-specific photo-ionization cross sections. By directly linking subaqueous light conditions, sediment concentration, and mineral-specific bleaching behaviour, our findings provide empirical quantification that can inform luminescence dating, provenance studies, and tracing of sediment transport in dynamic coastal systems.

**Supplementary Information:**

The online version contains supplementary material available at 10.1038/s41598-026-44245-6.

## Introduction

Luminescence signals in quartz and feldspar minerals are widely used in geosciences for dating e.g.,^1–5^ and sediment provenance studies e.g.,^6,7^. These signals record the last exposure of sediment grains to sunlight and are assumed to reset, or bleach, upon daylight exposure prior to deposition and burial^8^. Full resetting of the luminescence signal of interest in at least a portion of the grains is a prerequisite for luminescence dating e.g.,^9^. In contrast, partial resetting of signals may retain information about sediment transport pathways and histories and allow identifying sediment sources and pathways^10–13^. The big advantage compared to other tracing approaches is that all grains potentially can thus be used as tracer. Improved understanding of sediment pathways supports coastal management, like evaluating sediment dispersal after nourishment.

In subaqueous environments such as rivers, estuaries, and coastal settings, daylight exposure of sediment grains in transport is driven by water depth and turbidity^14–17^. Light intensity rapidly decreases with depth as the spectrum narrows due to preferential absorption of longer wavelengths (i.e., infrared) and scattering of UV and blue light. The latter typically penetrates deeper in clear water but attenuates more rapidly under high suspended sediment concentrations^17–21^. This greatly affects luminescence signal bleaching, as short wavelengths are most effective at resetting luminescence^21,22^. Spatial and temporal variability in subaqueous light conditions is high, leading to significant differences in bleaching behaviour across environments.

Previous laboratory studies have measured bleaching rates under controlled light sources^22–24^ and for several turbidity levels^15,16^. Some field studies have documented luminescence signal loss during subaqueous light exposure^21,25^, and more recently, simultaneously recorded light spectra^19^. However, none of these field studies have captured bleaching at single-grain level while also documenting underwater light spectra. Bulk signal measurements obscure the heterogeneity between individual grains^15,21^ and provide no information on the shape of the dose distribution, which offers insight into the mechanisms of the bleaching process.

The aims of this work are to: (1) determine luminescence resetting rates under water in natural conditions for a wide array of luminescence signals; (2) relate the bleaching rates to subaqueous light intensity and spectrum; (3) identify main controls on under water light intensity and spectrum. These aims are met through a one-day field experiment in the Ameland tidal inlet in the Dutch Wadden Sea where samples were exposed to light under water, while simultaneously monitoring light spectra and suspended sediment concentration as a function of depth. Our experiment provides the first direct measurements of single-grain luminescence signal loss under natural tidal conditions. The integrated approach provides understanding of luminescence bleaching in subaqueous environments, with potential implications for both luminescence dating and sediment tracing applications.

## Materials and methods

### Study site and experimental design

**Fig. 1:**
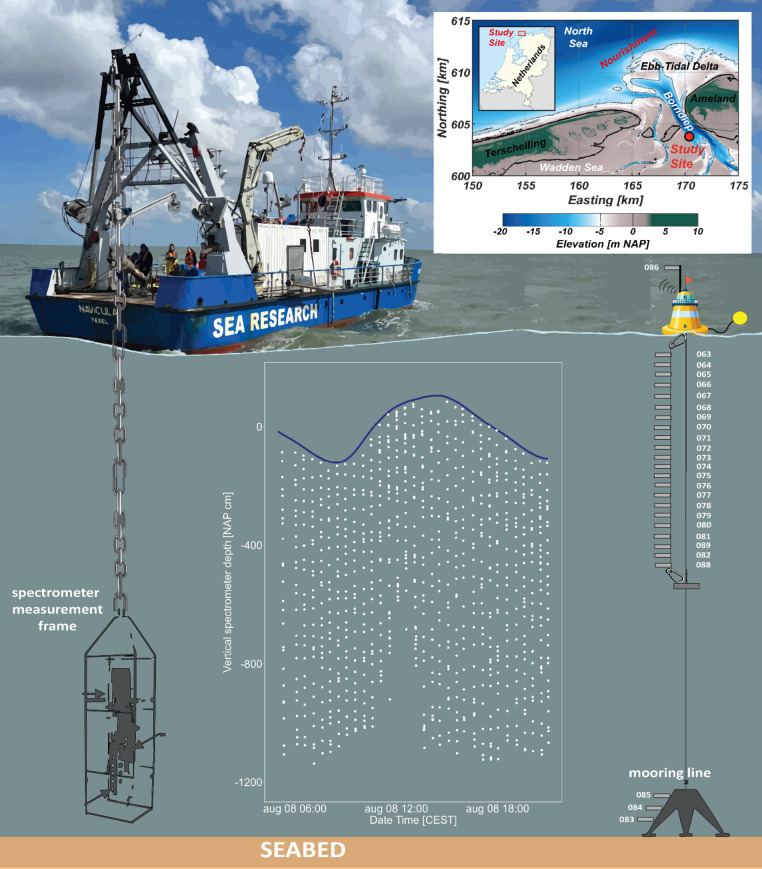
Experimental set-up at sea: frame with spectrometer and Optical Backscatter Sensor (OBS) hanging from the vessel’s stern (left) and mooring line with ETFE packages (right). The central inset shows the tidal water level fluctuations, and the datapoints for light spectra and suspended sediment concentration (SSC) collected during the experiment. The photograph of the RV Navicula by Z.Erdem, adapted and reproduced with permission. The inset map shows the bathymetry of Ameland Inlet, developed by the authors using MATLAB R2024a. Bathymetry source: Rijkswaterstaat Vaklodingen (CC0 1.0 Universal license). Elevation source: Actueel Hoogtebestand Nederland (AHN), Rijkswaterstaat (CC0 1.0 Universal license). The Netherlands overview map is licensed under the Creative Commons Attribution-ShareAlike 3.0 Unported (CC BY-SA 3.0) license and available at https://commons.wikimedia.org/wiki/File:Netherlands_location_map.svg.

The Ameland tidal inlet in the Dutch Wadden Sea (Fig. 1) is a meso-tidal, mixed-energy system shaped by ~ 2-meter tidal amplitudes and a moderate wave climate^26^. These conditions generate strong temporal and spatial variability in water depth and turbidity^27^. Turbidity at the distal ebb-tidal delta increases during storms and near low water slack, due to resuspension of fine sand and flocculated mud^28^. We conducted a field experiment from dawn to dusk covering over one full semi-diurnal tidal cycle (Fig. 1).

Purified quartz and feldspar mineral extracts were exposed to natural light conditions at different depths, by sealing grains in ETFE packages^19^, and attaching these to a vertical mooring line held taut by a 400 kg anchor and stabilized at the surface (Fig. 1). Samples with known dose (see Sect.  2.2) were transported in light-tight bags, and sunk into the water before dawn. Six CTD-divers (Van Essen Instruments) distributed along the mooring line, anchor, and buoy recorded pressure throughout the deployment, allowing actual sample depth to be monitored over the course of the measurement day. The Eulerian (fixed frame of reference) design aimed to submerge the samples at constant water depth, but due to strong currents the floating buoy was dragged down to 4.5 m depth during an interval of 3 h. At dusk, the mooring line with samples was retrieved and samples were sealed in light-tight packaging.

### Luminescence samples and measurements

After retrieval, 212–250 μm grains were analysed at the single-grain level using an EMCCD-based luminescence imaging system on a Risø TL/OSL DA-20 luminescence reader, enabling direct optically stimulated luminescence (OSL), thermoluminescence (TL), and infrared stimulated luminescence (IRSL) measurement per grain^29^. We selected this grain size because it matches the d_50_ of the sand in the tidal inlet of Ameland^27^ and is the standard fraction used for single-grain luminescence measurements^29^. Quartz and feldspar luminescence signals were measured using standard single aliquot regeneration (SAR) and multiple elevated temperature post-IR IRSL (MET-pIRIR) protocols, respectively. TL signals were recorded during the preheat step of the feldspar protocol. Equivalent doses were obtained using a central age model (CAM)^30^. A SAR-SARA test was conducted to test for uncorrected sensitivity changes in the first SAR measurement cycle for the MET-pIRIR protocol^31^. All luminescence-specific terminology and concepts are defined in the glossary provided in Supplement S1. Further details on the SAR-SARA test (Fig. S1), sample preparation, and measurement settings are provided in Supplement S2.

### Light spectra and bleaching potential

Under water light spectra were recorded from the vessel, about a few hundred meters away from the bleaching experiment to avoid disturbance. Spectral irradiance (320–950 nm) was measured using two TriOS RAMSES ACC-2 VIS spectrometers: one mounted on the vessel measuring above-water light and one on a measurement frame, enabling vertical profiling of the water column in ~ 20 cm steps (Fig. 1). In total, 32 profiles of spectral irradiance over depth were collected through the day (Fig. 1). The underwater spectrometer also recorded pressure, which was converted to inundation depth assuming an average sea water density of 1023.6 kg m^− 3^. Water level data measured at Terschelling Noordzee station^32^ enabled conversion of all measurements relative to NAP (Dutch ordnance level). To calculate total irradiance (in mW m^−2^), each spectrum was integrated over the full wavelength range. These values were interpolated across depth and time to produce a heatmap of total light intensity.

We also calculated expected luminescence bleaching based on subaqueous light exposure. Towards this, we combined the spectral data with mineral-specific photo-ionization cross-sections, available for quartz OSL and feldspar IRSL^33,34^. For each measured light spectrum, we calculated the exposure duration required to reset signals to 50% of their initial value. Additionally we calculated expected bleaching for quartz OSL and feldspar IRSL signals based on the total light exposure during the experiment. Detailed calculations are in Supplement S3.

### Suspended sediment concentration

Suspended sediment concentration (SSC) over the water column was derived from measurements conducted using a Campbell OBS-3 + sensor. The OBS measurements recorded the level of turbidity in the water column (in NTU), which was converted to SSC (g L^− 1^) following calibration. Details on the calibration procedures are provided in Supplement S4.

## Results

### Subaqueous luminescence signal resetting

**Fig. 2:**
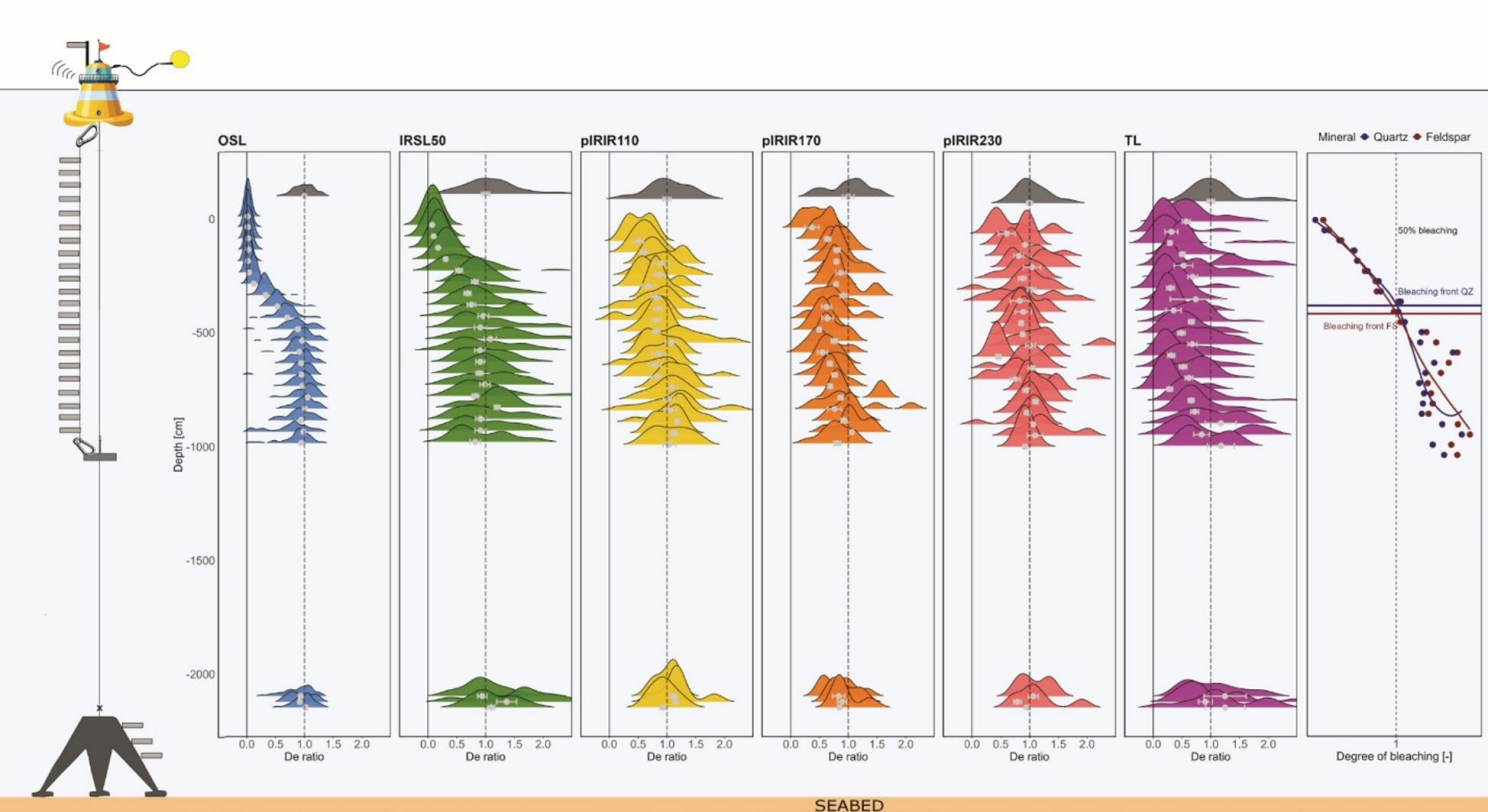
Single-grain equivalent dose density distributions for quartz OSL, four feldspar signals (IRSL50; pIRIR110; pIRIR170; pIRIR230), and a TL signal over depth. The Central Age Model (CAM) equivalent dose, expressed as ratio to starting dose, for each sample is indicated by a light grey dot where the whiskers indicate the model uncertainty. The dashed line marks a remnant/starting dose ratio of unity, indicating that no bleaching occurred. The solid line indicates complete bleaching. The outer right graph shows the degree of bleaching calculated from light spectra and photo-ionization cross-section data. The blue line marks the bleaching front for 50% bleaching of quartz OSL and the red line the 50% bleaching front of feldspar IRSL.

Single-grain equivalent-dose (D_e_) distributions of non-exposed samples are normally distributed for the quartz OSL signal, while IRSL, pIRIR and TL distributions are skewed (Fig. 2, grey distributions). The remnant-dose distributions obtained on light-exposed samples are all normalised to the central value of the D_e_ distribution for the non-exposed sample. Remnant-dose distributions obtained for samples exposed on the frame at about 20 m depth, are indistinguishable from the D_e_ distribution in unexposed samples. This indicates that no bleaching occurred at this depth and that light-exposure during sample installation (at dawn) and extraction (at dusk) did not cause significant bleaching.

Quartz OSL was fully reset for samples exposed in the upper 3 m of the water column, while negligible bleaching occurred below 5 m (Fig. 2, blue). In between, there is a relatively steep bleaching front, comparable with bleaching fronts reported in rock surface dating^35^. This pattern is reflected in the single-grain remnant dose distributions for individual samples, which show narrow, well-bleached quartz populations in the upper part of the profile, and increasingly overdispersed distributions at greater depth. The IRSL50 signal is nearly completely reset in the upper meter (Fig. 2, green), and unaffected below 5.5 m, with a less steep bleaching front than quartz OSL. The single-grain remnant dose distributions indicate some heterogeneity in bleaching. In line with expectations e.g.,^22^, the pIRIR110, pIRIR170 and pIRIR230 signals are less reset and are only (partially) bleached in the upper 50 cm of the water column (Fig. 2, yellow, orange, pink, respectively). Surprisingly, the TL signals (Fig. 2, purple), expected to be least bleachable^36,37^, appear to be partially reset even down to 10 m depth.

### Subaqueous light spectra, SSC and luminescence bleaching rate

**Fig. 3:**
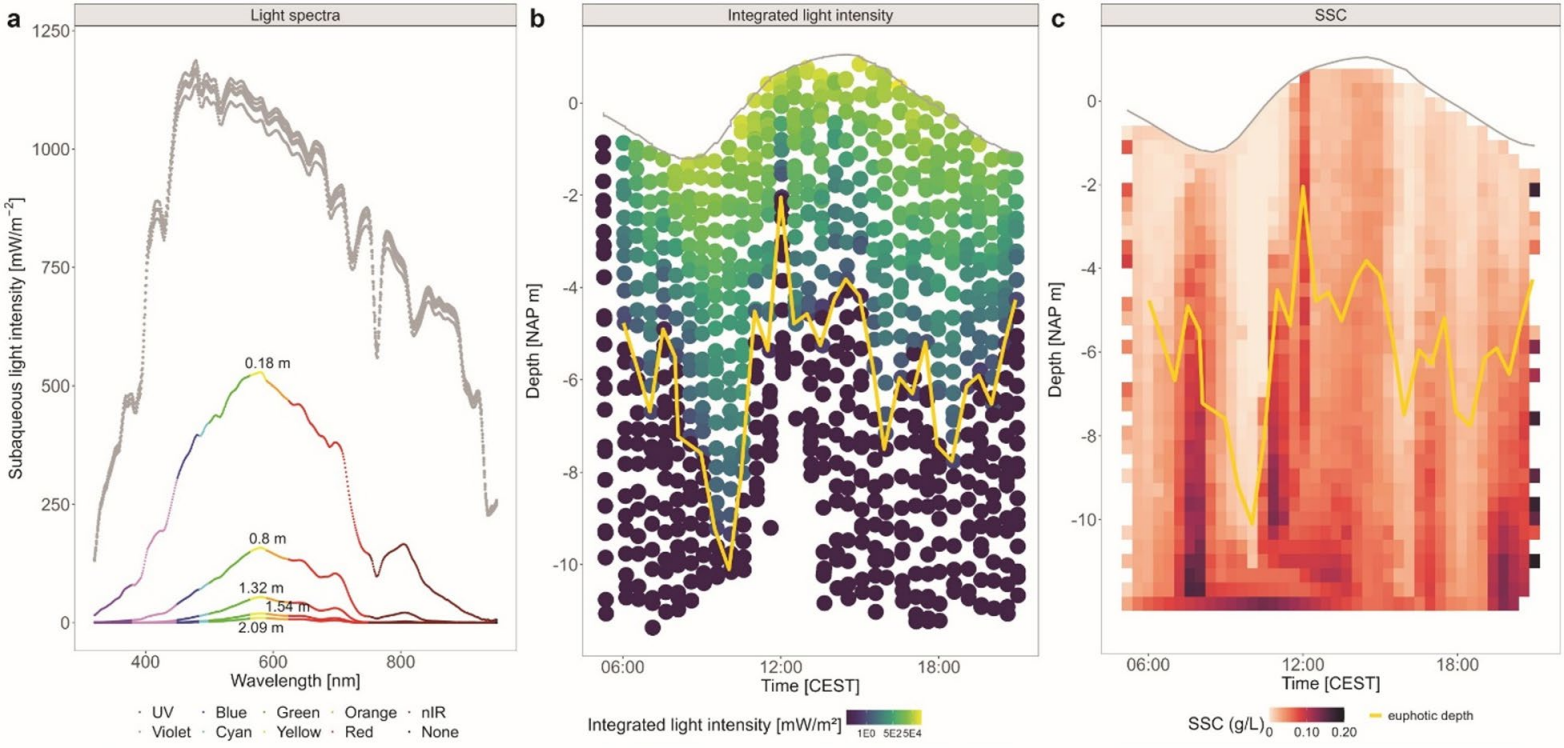
(**a**) Example of light spectra measured above (grey) and below (coloured) the surface during flood at 3 PM CEST showing light attenuation with depth. (**b**) Light intensity integrated over the full spectrum for each datapoint. (**c**) Heatmap of SSC data. The scale highlights low (white) to high (dark red/ purple) SSC concentrations in g/L. The euphotic depth is indicated with the yellow line.

**Fig. 4:**
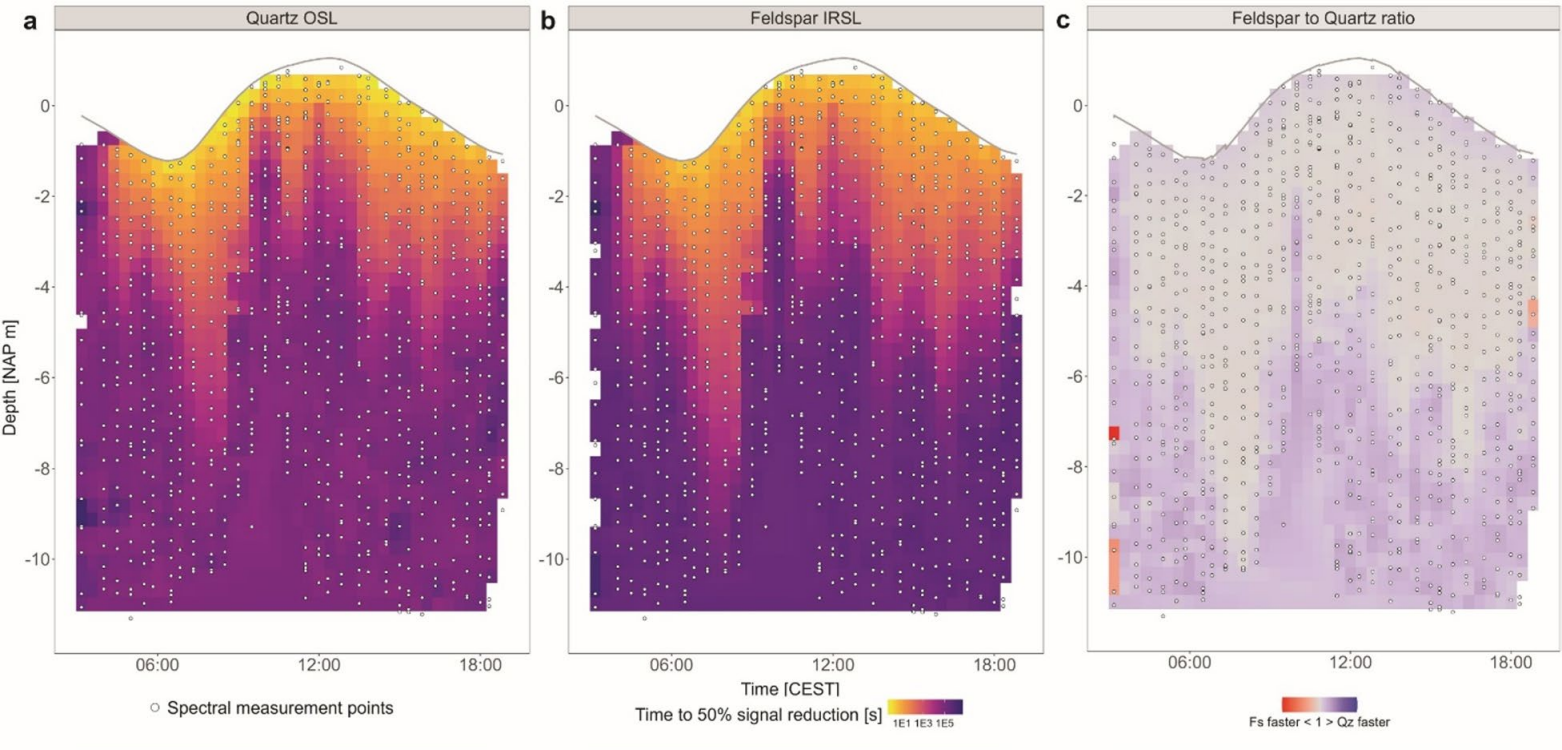
Heatmaps of depth- and time-resolved durations to reset luminescence signals by 50% for quartz OSL (**a**) and feldspar IRSL (**b**). (**c**) Ratio of feldspar to quartz bleaching durations to reset luminescence signals by 50%.

Light intensity rapidly decreases with depth, as expected (Fig. 3a,b). Our spectral measurements show that high-energy short wavelengths (blue-green), which typically penetrate the deepest in clear water, attenuate more rapidly here due to suspended sediment scattering Fig. 4^15^. At the same time, low-energy wavelengths (red and near-infrared) also exhibit strong attenuation, primarily due to absorption by water. Overall, attenuation patterns vary throughout the day in response to changes in incoming solar radiation (Fig. 3b) and turbidity (Fig. 3c).

The SSC profiles reveal elevated sediment concentrations during ebb tide (Fig. 3c), especially in the morning when currents flow from the Wadden Sea basin toward the North Sea. These higher concentrations strongly reduce subaqueous light penetration, as demonstrated by the inverse relationship between SSC and euphotic depth (yellow line in Fig. 3b,c). Importantly, this attenuation is wavelength-dependent: high-energy blue-green light is disproportionally reduced under turbid conditions. The complete set of light spectra and SSC across all profiles, depths, and tidal stages is included as animated GIFs in Supplement S3.

At greater depths, reduced light levels lead to significantly longer exposure times needed to reset luminescence signals. Heatmaps of the calculated time required to reduce luminescence signals by 50% show that quartz resets within ~ 10 s in the upper 50 cm at noon (Fig. 4a), while feldspar IRSL needs a few tenths of seconds in this interval (Fig. 4b). For both minerals, bleaching durations to reset signals by 50% increase rapidly with depth (Fig. 4a,b). To directly compare the bleaching behaviour of the minerals, we calculated the ratio of the duration for 50% reduction of the quartz OSL versus feldspar IRSL signal (Fig. 4c). A clear pattern emerges: near the surface, quartz OSL bleaches fastest (purple hues), while between approximately − 2 and − 5 m depth, feldspar IRSL requires less time for 50% signal reduction (red hues). At deeper depth, where bleaching is slow for both signals due to low light intensity, quartz OSL is again the fastest bleaching signal. Animated GIFs showing 50% bleaching duration curves for both quartz OSL and feldspar IRSL across tidal stage are provided in Supplement S3.

Expected luminescence signal resetting calculated from light exposure and optical cross section, predicts a bleaching front (50% resetting) to occurs at ~ 3.5–4 m depth for both minerals (Fig. 2, right). Given the assumptions made in this calculation, the experimental challenges, and the need for additional photo-ionization cross-section data for uncertainty analysis, the agreement with observed bleaching front depths (Fig. 2) is highly encouraging.

## Discussion

### Effect of tides and sediment concentration on subaqueous luminescence bleaching

The SSC varies over the tidal cycle (Fig. 3c), with highest concentrations during ebb tide when currents are outward from the Wadden Sea basin toward the North Sea. This finding is consistent with the ejection of fine sediment from the Wadden Sea. Flood tide SSC remains comparatively low throughout the profile, consistent with advection of less turbid water from the North Sea^28,38,39^. However, early in the flood stage SSC tends to be higher, likely reflecting remnant resuspension or early flood-driven advection of turbid water masses. These tidal variations in SSC strongly influence light attenuation (Fig. 3b) and, as a result, the calculated durations required to reset luminescence signals by 50% (Fig. 4a, b).

During ebb tide, elevated SSC, particularly between 4 and 10 m depth, result in pronounced light attenuation (Fig. 3b) and consequently longer bleaching durations at depth (Fig. 4a). In contrast, after the tidal turn, as SSC declines as clearer water flows in^28^, light penetrates more deeply and bleaching durations shorten significantly. These patterns highlight how tidal modulation of SSC governs underwater light availability and thus controls when and where luminescence signals in natural environments reset.

SSC also governs ratios of bleaching duration of quartz OSL and feldspar IRSL. The cross-over depth at which feldspar IRSL bleaches faster than quartz OSL is about − 5 m in clear water and decreases to -2 m in more turbid water. This crossover reflects the interplay between the underwater light spectrum (Fig. 3a,b) and the minerals’ wavelength-dependent photo-ionization cross-sections (Fig. S2). Quartz OSL bleaches faster than feldspar IRSL in response to UV and blue light, which dominate near the surface but attenuate rapidly with depth (Figs. 3a and 4a). Yet, feldspar IRSL bleaches faster than quartz OSL for longer wavelengths (visible to near-infrared), and continues to bleach in deeper water where quartz receives insufficient energy (Figs. 3a and 4b). It should be noted that feldspar bleaching durations presented here refer to the IRSL signal only. Absence of information on wavelength specific photo-ionization cross-section for pIRIR signals precluded us from similar analyses for those signals.

The same hydrodynamic processes that cause sediments to be suspended and reduce light penetration, also disperses grains used for luminescence dating into the water column. The grain size of 212–250 μm often used for luminescence analysis is more likely to be transported as bedload close to the bed^28^. Only during highly energetic events, these grains are brought in suspension and may end up higher in the water column. However, these will also be circumstances with high turbidity, reducing bleaching opportunities.

### Observed and calculated luminescence resetting

Quartz OSL signals are fully reset in the upper ~ 3 m, with a steep bleaching front between ~ 3 and 5 m and minimal resetting below 5 m during our one-day experiment. This pattern reflects the strong sensitivity of quartz to short-wavelength UV-blue light, which drives rapid signal loss in the upper layers before it significantly attenuates with depth (Fig. 3a,b). Single-grain distributions (Fig. 2) reinforce the depth trends: quartz grains show tight, well-bleached populations in the upper layers. The sharp transition in the quartz profile mirrors bleaching fronts observed in rock surface dating^35^, underscoring the importance of spectral light penetration in governing luminescence signal resetting.

Feldspar IRSL50 signals exhibit a more gradual bleaching front, nearly fully reset in the upper 1 m and unaffected below ~ 4.5 m. pIRIR110, pIRIR170, and pIRIR230 signals show only limited bleaching in the upper 0.5 m, in line with their reduced bleachability^22^. The single-grain distributions (Fig. 2) reinforce these trends, especially the pIRIR signals display broader and more mixed distributions across depth, consistent with heterogeneous resetting, likely related to grain-to-grain differences in bleachability of grains^40^. TL signals unexpectedly indicate partial resetting down to ~ 10 m depth, suggesting the presence of a TL component that is more light-sensitive than the quartz OSL and felspar IRSL signals. Clearly this requires further investigation.

Observed bleaching patterns align well with expected bleaching calculated from time-integrated observations of underwater light spectra and mineral-specific photo-ionization cross-section data (Fig. 2). Overall agreement between calculated and observed bleaching is encouraging, especially in the light of several limitations for calculating expected bleaching based on measured light spectra. We based our calculations on 50% signal reduction assuming a linear relationship of signal reduction with light exposure, which is clearly a simplification. Moreover, the photo-ionization cross-sections are based on a single specimen, while both quartz and feldspar luminescence properties are known to be variable depending on e.g., provenance e.g.,^6^. It is highly likely that this is also true for photo-ionization cross sections. Photo-ionization cross-section data are currently unavailable for feldspar pIRIR and TL signals, preventing direct comparison of observed and calculated bleaching for those signals.

### Implications for luminescence dating, tracing and coastal management

Our findings underscore the need to account for mineral-specific bleaching kinetics when applying luminescence dating in subaqueous settings^41^. Effective dating requires that grains are transported or deposited at relatively shallow depths where sufficient light is available for bleaching^42^. Longer transport will increase both potential duration of light exposure, and chances for grains to be exposed close to the water surface, explaining improved resetting in downstream direction of rivers^11,14^. At our site, resetting is rapid in the upper metres, but very long exposures would be needed at greater depth. Given that grains in the sizes used for our experiment (212–250 μm) are not typically transported in suspension, bleaching is more likely to occur in shallow coastal areas outside main tidal channels^43^.

The observed incomplete resetting of all luminescence signals underscores their value as indicators of sediment transport and exposure history. Comparison of signals of different bleachability, ideally from a single grain, retain a measurable imprint of subaqueous light exposure during transport. In particular, pIRIR signals offer potential for tracing sediment pathways and residence times given their stability and resistance to rapid resetting. This principle underpins recent advances in luminescence tracing approaches^10,13,14^, which exploit the incomplete resetting of luminescence signals to reconstruct sediment provenance, transport distance, or reworking intensity. Our results affirm that signal preservation is not merely a dating challenge, but a useful feature for tracing sediment movement through light-limited environments. However, we acknowledge that several additional steps will be needed to develop our Eulerian approach of signal resetting at fixed depth under moderate conditions into a Lagrangian approach to model light exposure and luminescence signal resetting during grain transport in a dynamic coastal environment. These additional steps are required to fully exploit the potential of luminescence tracing methods.

Beyond luminescence applications, our findings carry broader implications for coastal management. Sediment availability is a critical factor in successful wetland restoration^44^, while the phenomenon of coastal squeeze, where human pressures and sea-level rise restrict the natural space for sediment redistribution, increasingly threatens coastal resilience^45^. At global scale, suspended particle matter in coastal waters is declining due to human interventions and reduced riverine sediment delivery^46^. These trends highlight the urgency of understanding sediment pathways and reworking processes. Existing tools, such as Lagrangian particle tracking models^45^, provide valuable simulations of sediment dynamics but rely on assumptions that are challenging to validate in the field. By quantifying in-situ bleaching in terms of signal loss (Fig. 2) and duration (Fig. 4), our study provides empirical constraints that can be linked to and tested with model-based approaches.

## Conclusions

This study explores how tidal dynamics and suspended sediment concentrations influence the underwater light climate, affecting the extent and rate of luminescence signal resetting. By combining in-situ measurements of signal resetting in a tidal inlet with bleaching durations calculated from spectral light data and mineral-specific photo-ionization cross-sections, we demonstrate that bleaching is highly sensitive to water clarity and depth. While quartz OSL resets rapidly near the surface, its resetting is incomplete at depth where much of the sediment transport occurs. Feldspar signals, particularly pIRIR, exhibit even greater resistance to resetting. Our findings provide empirical constraints on the use of luminescence dating in dynamic, light-limited settings by highlighting the importance of signal-specific bleaching behavior. While partial bleaching of quartz and feldspar luminescence signals may be a hindrance to dating applications, it opens new avenues for reconstructing sediment pathways and exposure histories in coastal and fluvial systems.

## Supplementary Information

Below is the link to the electronic supplementary material.

Supplementary Material 1

## Data Availability

All data, R scripts, and code required to reproduce the analysis and figures in this study are published in full through the 4TU.ResearchData repository, including a README file detailing file structure and instructions for use. The dataset has been assigned a DOI and made publicly available at the time of publication, DOI: https://doi.org/10.4121/f4b0d921-5c7d-49f8-abd1-5d13169139db.
