# Supplementary material for: Resetting of quartz and feldspar luminescence signals under water
Source: Sci Rep. 2026 Mar 17;16:13735. doi: 10.1038/s41598-026-44245-6 (PMC13125625; doi:10.1038/s41598-026-44245-6)
Supplement: Supplementary file 1 — Supplementary Material 1 [file 41598_2026_44245_MOESM1_ESM.docx]

­­Supporting Information for

**Resetting of quartz and feldspar luminescence signals under water**

Anna-Maartje de Boer^1*^, Natascia Pannozzo^2^, Stuart G. Pearson^2^, Tjitske J. Kooistra^3^, Bram van Prooijen^2^ and Jakob Wallinga^1^

^1^Soil Geography and Landscape Group & Netherlands Centre for Luminescence Dating, Wageningen University & Research, Droevendaalsesteeg 3, 6708 PB Wageningen, the Netherlands.

^2^Faculty of Civil Engineering & Geosciences, Delft University of Technology, Stevinweg 1, 2628 CN Delft, the Netherlands.

^3^Department of estuarine and Delta Systems, Royal Netherlands Institute for Sea Research, Korringaweg 7, 4401 NT Yerseke, the Netherlands.

*Corresponding author: Anna-Maartje de Boer (anna-maartje.deboer@wur.nl)

Contents

[S1 Introduction and glossary 3](#_Toc207022329)

[S2 Luminescence sample preparation and measurement protocols 5](#_Toc207022330)

[Figure S1. SAR-SARA test on sample NCL-1123090 for four feldspar signals. 6](#_Toc207022331)

[Table S1. Adopted MET-pIRIR measurement protocol for the feldspar fraction. 7](#_Toc207022332)

[Table S2. Adopted SAR measurement protocol for the quartz fraction. 7](#_Toc207022333)

[S3 From light spectra and bleaching rates to exposure duration required to reset signals by 50% 8](#_Toc207022334)

[Table S3. Tabular overview profile measurements. 9](#_Toc207022335)

[Figure S2. a) Photo-ionization cross-section data, digitized from (Spooner 1994a, Spooner 1994b). b) Bleaching sensitivities curves were derived by inverting these energy values. 11](#_Toc207022336)

[S4 Turbidity calibration 11](#_Toc207022337)

[Figure S4. Suspended Sediment Concentration (SSC) profiles. 13](#_Toc207022338)

[References 13](#_Toc207022339)

S1 Introduction and glossary

This supplementary information file provides additional details supporting the main manuscript. It includes a glossary of luminescence-specific terminology (Supplement S1), detailed protocols on luminescence sample preparation and measurement (S2), a stepwise explanation of how light spectra and mineral-specific detrapping sensitivities were used to derive bleaching durations (S3), and methods and calibration results for suspended sediment concentration (S4).

In addition to this document, we provide a set of animated GIFs visualizing:

1. Subaqueous and subaerial light spectra over depth and tidal stage
2. Calculated bleaching efficiency curves for quartz OSL and feldspar IRSL

The complete data, code and GIFs will be published open access via the 4Tu.ResearchData repository to enhance reproducibility and transparency. This will include a detailed readme file outlining the data structure, file formats, and instructions to reproduce key figures and calculations.

**Luminescence glossary:**

- *Anomalous fading*: describes the loss of IRSL signal over time at ambient temperatures due to quantum-mechanical tunneling of trapped electrons. It affects feldspar signals and can lead to age underestimation if uncorrected ^1,2^.
- *Bleaching*: refers to the process by which trapped electrons in mineral grains are released through exposure to light, effectively resetting the luminescence signal. Only grains for which the luminescence signal of interest is fully bleached (reset) upon deposition, may provide an accurate account of burial age ^3^.
- *Central Age Model (CAM)*: the CAM is a statistical model used to calculate a representative palaeodose from a distribution of individual equivalent dose estimates. It assumes a log-normal distribution of doses and is particularly suited to well-bleached populations ^4^.
- *Electron Multiplying Charge-Coupled Device (EMCCD)*: is a highly sensitive imaging sensor used in single-grain luminescence systems to detect low-intensity luminescence from individual grains. It enables spatially resolved signal detection and grain-specific dose estimation ^5,6^.
- *Equivalent dose (D_e_) distribution*: represents the spread of equivalent doses, measured across multiple grains or aliquots. It reflects the variability in signal resetting and ionizing radiation exposure among grains.
- *InfraRed Stimulated Luminescence (IRSL)*: is a signal measured from feldspar minerals by stimulating with infrared light, which releases electrons from light-sensitive traps. The K-feldspar IRSL signal saturates at higher doses than quartz OSL but is often affected by anomalous fading ^7^.
- *Optically Stimulated Luminescence (OSL)*: is a luminescence signal emitted by minerals such as quartz when they are stimulated with typically blue or green light, releasing charge from traps and emitting photons. The quartz OSL signal is usually the luminescence signal of choice for dating sediments, as the signal is quickly reset upon light exposure and stable over geological timescales ^8^.
- *post-IR IRSL (pIRIR)*: refers to feldspar luminescence signals measured after an initial IR stimulation at lower temperature, typically using elevated temperature IR stimulation. In comparison with IRSL, pIRIR signals are less affected by anomalous fading, but also less likely to be fully reset prior to deposition and burial due to lower sensitivity to (day)light ^9^.
- *Remnant dose distribution*: refers to the range of remaining luminescence doses in grains after natural or experimental light exposure. It provides insight into heterogeneous bleaching processes in depositional environments.
- *Single-Aliquot Regeneration (SAR) protocol*: a widely used procedure in luminescence dating where a single aliquot of grains is repeatedly stimulated and irradiated in cycles to construct a dose-response curve. This method accounts for sensitivity changes and enables accurate determination of the equivalent dose ^10^.
- *Single-Aliquot Regeneration – Single-Aliquot Regeneration Additive (SAR-SARA) protocol*: a test used to detect and correct for sensitivity changes that may occur during the SAR measurement cycle. It compares regenerated signals with additive dose responses to improve accuracy in dose reconstruction ^1^.
- *Thermoluminescence (TL)*: a luminescence signal emitted when minerals are heated ^11^. It provides an alternative to light-stimulated techniques and can be used to assess bleaching history and signal composition. TL signals are expected to be less light sensitive compared to IRSL and OSL signals.

S2 Luminescence sample preparation and measurement protocols

Feldspar grains for our analysis were extracted from samples obtained from Brown Bank sediment in the Dutch North Sea ^12^. We chose to work on a naturally dosed sample rather than an irradiated sample to avoid heterogeneous irradiated samples ^13^. A K-rich feldspar extract was prepared using wet sieving (212–250 μm), chemical treatments (10% HCl and H₂O₂), and density separation (2.58 g cm^-^³). Three samples with identical palaeodose were combined (4.6 g total). Feldspar was measured using a MET-pIRIR protocol (IRSL₅₀, pIRIR₁₁₀/₁₇₀/₂₃₀) (Table S1). All signals were detected using a BG-3 + BG-39 (3.0 + 4.0 mm) blue filter package centered on 410 nm emission. A total of 270 channels were recorded over 108 s (0.4 s per channel), with stimulation during the from channel 11 onwards. Signal integration was chosen based on EMCCD-specific recommendations of de Boer et al. (2024) and therefore performed over the first 8 s (channels 11–31), with background subtraction over the last 20 s (channels 210–260). TL was recorded in 250 channels during the preheat while heating to 250 °C. The TL signal was integrated over the main peak area (channels 114-250; 114-250 °C), a background level was taken pre-peak (channels 1-57).

A Single Aliquot Regeneration and Added dose (SAR-SARA) test was performed for the MET-PIRIR protocol to see if there were any sensitivity changes between the natural signal and first test dose ^14^. Aliquots were first prepared from sample NCL-1123090 and split into four batches of three aliquots each. The second, third, and fourth batch were given additional beta dose on top of the natural dose of 25, 50, and 100 Gy, respectively. All batches were then measured using the full SAR protocol to determine the natural dose (batch 1) and the natural plus added beta doses (batch 2-4). A trendline is fitted through this data for each of the signals (IRSL_50_, pIRIR_110_, pIRIR_170_ and pIRIR_230_). In absence of trapping sensitivity change, the slope of these lines should be unity. All trendlines are indeed within 10% of this ideal value (Figure S1).

For the exposure experiment of quartz, we used 4.1 g of Risø calibration quartz (batch 200). No additional chemical and or physical treatments were necessary to perform. Quartz signals were measured using a Hoya U-340 (2.5 mm) in combination with a coated UG-11 (2.0 mm) filter to block the 720 nm breakthrough. Quartz OSL was measured at 125 °C using a standard SAR protocol with late-background subtraction (Table S2). A total of 250 channels over 20 s (0.08s per channel) were recorded. Signal integration was performed over the first 1.6 s (channels 1-20), with background
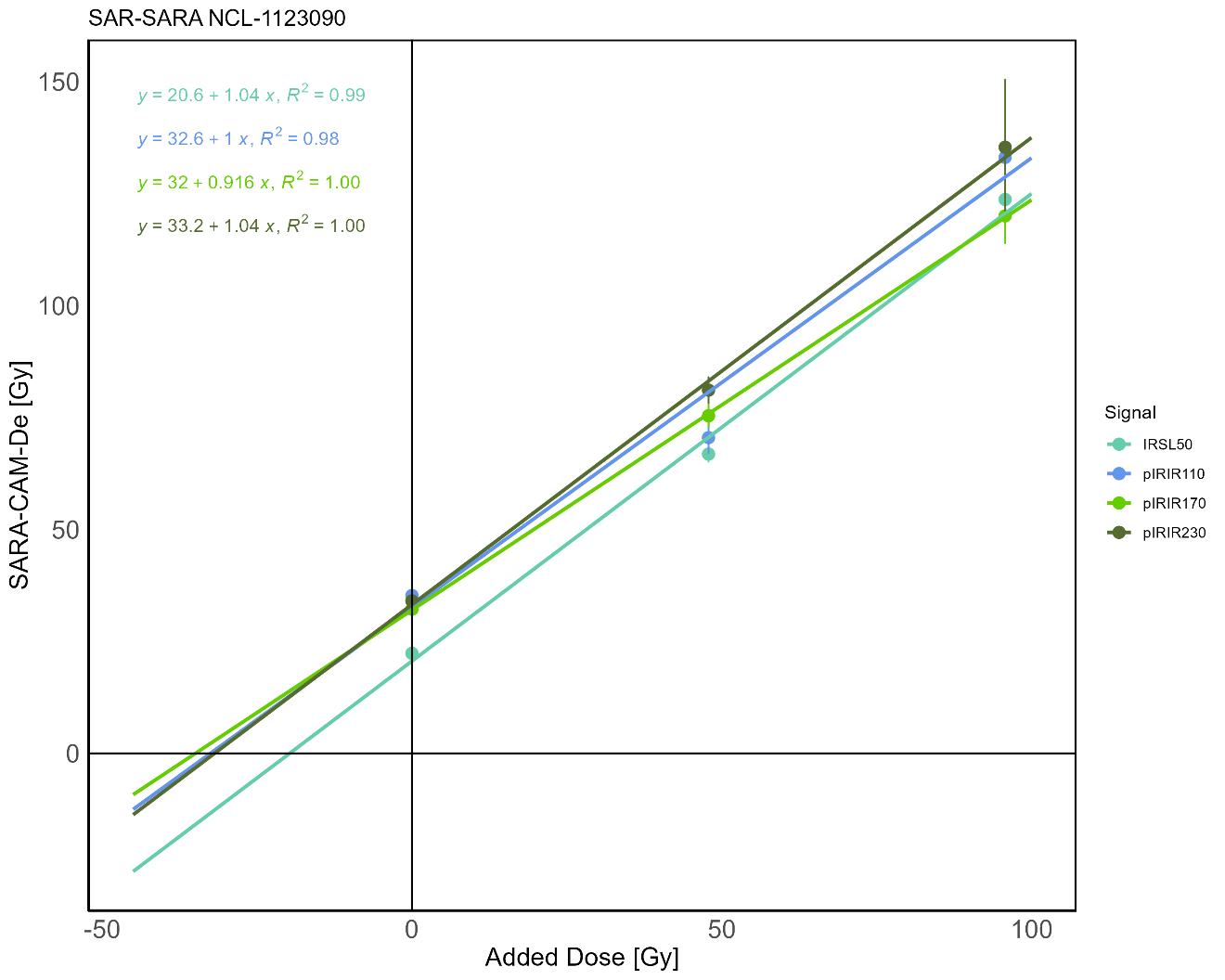
subtraction over the last 4 s (channels 200-250).

Figure S1. SAR-SARA test on sample NCL-1123090 for four feldspar signals.

After retrieving samples following the full-day exposure sediment, three single-grain discs were prepared for each mineral (300 grains). All measurements were performed with an EMCCD system ^5^ and data were integrated for a 450 μm region of interest with the use of Risø Viewer+ software. This ROI has been shown to provide a good compromise to enhance signal detection while minimizing crosstalk^15^. Dose-response curves were fit using single saturating exponential functions. Rejection thresholds for recycling, recuperation relative to R1, and D_e_ uncertainty were set at 20%, as a slightly broader criterion than usual is appropriate for bleaching studies to allow more grains to be accepted and thereby prevent the loss of grains that inherit information on bleaching. All data analysis was performed with the R-luminescence package ^16^.

| Step | Action | Signal |
| --- | --- | --- |
| 1 | Natural/regenerative dose (0, 7.6, 15.3, 30.1, 61.0, 0, 7.6 Gy) |  |
| 2 | Preheat at 180°C (60 s) |  |
| 3 | TL at 250°C (60 s) | TL-250 Li |
| 4 | IRSL at 50°C (100 s) | IRSL-50 Li |
| 5 | IRSL at 110°C (100 s) | pIRIR-150 Li |
| 6 | IRSL at 170°C (100 s) | pIRIR-225 Li |
| 7 | IRSL at 230°C (100 s) | pIRIR-290 Li |
| 8 | Warm bleach with IR-LEDs at 230°C (100 s) |  |
| 9 | Test dose (8.1 Gy) |  |
| 10 | Preheat at 180°C (60 s) |  |
| 11 | TL at 250°C (60 s) | TL-250 Ti |
| 12 | IRSL at 50°C (100 s) | IRSL-50 Ti |
| 13 | IRSL at 110°C (100 s) | pIRIR-150 Ti |
| 14 | IRSL at 170°C (100 s) | pIRIR-225 Ti |
| 15 | IRSL at 230°C (100 s) | pIRIR-290 Ti |
| 16 | Hot bleach with IR-LEDs at 260°C (100 s) |  |

Table S1. Adopted MET-pIRIR measurement protocol for the feldspar fraction.

| Step | Action | Signal |
| --- | --- | --- |
| 1 | Natural/regenerative dose (0, 4.3, 6.5, 8.6, 0, 4.3, 4.3 Gy) |  |
| 2 | Preheat at 220°C (10 s) |  |
| 3 | *IRSL at 30°C (60 s) |  |
| 4 | OSL at 125°C (20 s) | OSL-125 Li |
| 5 | Test dose (2.2 Gy) |  |
| 6 | Preheat at 220°C (10 s) |  |
| 7 | OSL at 125°C (20 s) | OSL-125 Ti |
| 8 | Hot bleach with blue LEDs at 230°C (20 s) |  |
| *Check for feldspar contamination only during last regenerative run | | |

Table S2. Adopted SAR measurement protocol for the quartz fraction.

S3 From light spectra and bleaching rates to exposure duration required to reset signals by 50%

Spectral irradiance was profiled at ~20 cm depth intervals using a submerged TriOS RAMSES ACC-2 VIS sensor, while an above-water reference spectrometer captured incident light, both in mW ^m-2^. In total 32 profiles were captured (Table S3). For each tidal stage, the spectral data from both sensors (subaerial and subaqueous) are visualized in animated GIFs, which are attached in two versions: (1) raw measurement values (“subaqueous” folder) and (2) values normalized to the subaerial signal (“subaerial” folder). File names follow the format ProfileX_subaqueous_spectra.gif and ProfileX_normalized_spectra.gif, where X refers to the tidal stage as indicated in Table S3 and can be found on the 4Tu.ResearchData repository.

| Tidal stage | Start time (UTC) | End time (UTC) | ID number | # of spectra |
| --- | --- | --- | --- | --- |
| 1E | 03:14:49 | 03:21:35 | 1-25 | 25 |
| 2E | 04:00:58 | 04:08:16 | 26-52 | 27 |
| 3E | 04:30:05 | 04:35:39 | 53-77 | 25 |
| 4E | 05:00:51 | 05:07:57 | 78-105 | 28 |
| 5E | 05:29:25 | 05:38:29 | 106-134 | 29 |
| 6E | 05:58:45 | 06:06:31 | 135-162 | 28 |
| 7E | 06:29:33 | 06:36:03 | 163-191 | 29 |
| 8E | 06:58:04 | 07:04:44 | 192-219 | 28 |
| 9LW | 07:28:43 | 07:35:42 | 220-248 | 29 |
| 10F | 08:01:39 | 08:07:16 | 249-277 | 29 |
| 11F | 08:30:02 | 08:35:24 | 278-305 | 28 |
| 12F | 08:59:56 | 09:05:20 | 306-335 | 30 |
| 13F | 09:29:51 | 09:36:57 | 336-368 | 33 |
| 14F | 09:59:56 | 10:05:26 | 369-396 | 28 |
| 15F | 10:29:50 | 10:34:54 | 397-422 | 26 |
| 16F | 10:59::43 | 11:09:51 | 423-453 | 31 |
| 17F | 11:29:08 | 11:39:34 | 454-487 | 34 |
| 18F | 12:02:13 | 12:09:11 | 488-516 | 29 |
| 19F | 12:28:06 | 12:34:35 | 517-542 | 26 |
| 20F | 12:59:28 | 13:06:09 | 543-569 | 27 |
| 21F | 13:30:04 | 13:36:30 | 570-596 | 27 |
| 22HW | 13:55:38 | 14:01:27 | 597-621 | 25 |
| 23E | 14:30:16 | 14:38:24 | 622-650 | 29 |
| 24E | 15:00:08 | 15:08:50 | 651-681 | 31 |
| 25E | 15:29:53 | 15:36:29 | 682-714 | 33 |
| 26E | 15:56:14 | 16:02:13 | 715-745 | 31 |
| 27E | 16:29:56 | 16:35:25 | 746-775 | 30 |
| 28LW | 17:03:31 | 17:08:23 | 776-800 | 25 |
| 29F | 17:31:27 | 17:37:33 | 801-831 | 31 |
| 30F | 18:00:29 | 18:05:58 | 832-859 | 28 |
| 31F | 18:29:45 | 18:37:12 | 860-891 | 32 |
| 32F | 18:55:16 | 19:00:49 | 892-918 | 27 |
| END | 19:56:46 | 19:56:46 | 919 | 1 |

Table S3. Tabular overview profile measurements.

To estimate durations required to reset signals by 50% and estimate a theoretical bleaching front after the full-day experiment, we combined mineral-specific bleaching sensitivities for quartz OSL and feldspar IRSL with the measured spectral irradiance data (Table S3). The calculation followed a three-step procedure:

***1. Deriving wavelength-specific bleaching sensitivity***

Previously published photo-ionization cross-section data ^17,18^ reports wavelength-specific energy (in J m^-^² nm^-1^) required to reset quartz OSL and feldspar IRSL signals to specific percentages: 10, 50, and 90% for feldspar IRSL and 5, 50, and 95% for quartz OSL (Fig. S2a). For our analysis, we used the 50% reduction values as these are provided for both minerals. Wavelength-specific bleaching sensitivity was then calculated as the reciprocal of the energy (in J m^-^² nm^-1^), yielding values in units of m² J^-1^ nm^-1^ (Fig. S2b).

***2. Calculating bleaching rates and bleaching duration***

This wavelength-specific bleaching sensitivity (in m² J^-1^ nm^-1^) was multiplied by the measured downwelling spectral irradiance (in W m^-^² nm^-1^) for each timepoint and depth (with 1 W = 1 J s^-1^). This product yields wavelength-specific bleaching rates with units of s⁻¹. Animated GIFs of the wavelength-specific bleaching rates are provided for all 32 tidal stages. Each GIF shows how wavelength-specific bleaching rates of quartz and feldspar change with depth, plotted together in the same graph (quartz in blue, feldspar in red). These files can be found in the folder named bleaching rates on the 4Tu.ResearchData repository and follow the naming convention bleaching_rates_50pct_data_StageX.gif, where X refers to the tidal stage in Table S3. These wavelength-specific bleaching rates are specific to each mineral (quartz or feldspar), time, and depth (Table S3), and were used for further integration over wavelength to calculate the effective light exposure under natural conditions. For quartz we integrated between 320 and 860 nm, and for feldspar between 320 and 950 nm, based on the range of available photo-ionization cross-section data (Fig. S2a). For convenience in all figures in the main manuscript, we expressed this effective light exposure as exposure duration required to bleach the luminescence signal by 50%. This exposure duration required to bleach was calculated by taking the inverse of the effective light exposure for 50% bleaching, i.e., by dividing ln(2) by the effective light exposure.

***3.Calculating the theoretical bleaching front***

To estimate the theoretical depth of the bleaching front after the full-day light-exposure experiment, we took the following steps. First, we calculated the wavelength specific light exposure during the experiment, by integrating spectral irradiance measurements over the day (J m^-^² nm^-1^). Towards this, we used depth intervals of 0.5 m, combining spectral irradiance measurements available within this interval. Then we multiplied these spectra with the wavelength-specific bleaching sensitivities (m² J^-1^ nm^-1^) (based on 50% signal loss) (Fig. S2b). Subsequently, we integrated the resulting curves across all wavelengths, yielding the effective light exposure relative to the light exposure required to reduce the signal by 50%. Hence, a value of 1 indicates sufficient light exposure to reset the signal by 50%, which indicates the approximate depth of the bleaching front after our one-day experiment. A value of zero indicates no bleaching and a value higher than 1 indicates less than 50% bleaching.


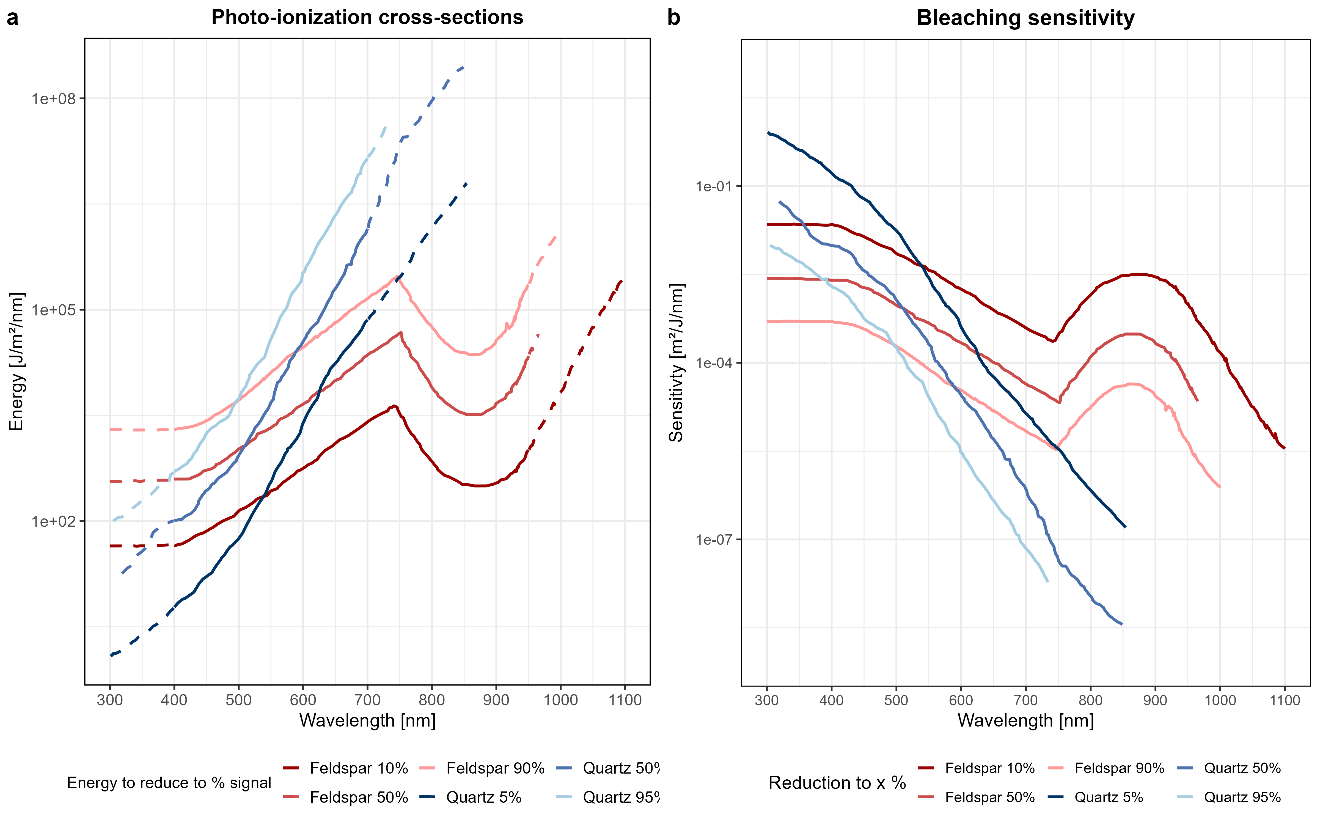
Figure S2. a) Photo-ionization cross-section data, digitized from ^17,18^. b) Bleaching sensitivities curves were derived by inverting these energy values.

S4 Turbidity calibration

Turbidity (NTU) was recorded using a Campbell OBS-3+ sensor and converted to suspended sediment concentration (SSC, in g/L) via a calibration experiment ^19^. The OBS sensor was suspended in a 16 L bucket filled with seawater and wet sediment samples from the Ameland Inlet surface seafloor through gravity coring. The bucket was equipped with a stirrer to keep the sediment in suspension and the OBS was attached to a frame, so that the measurement probe was facing downward at the center of the bucket, just below the water surface of the bucket. After each OBS measurement, the sediment concentration was diluted by removing half of the water-sediment mix volume and filling the bucket again with more filtered seawater. The first 100-500 mL of the water collected during each dilution was filtered over pre-weighed and dried GG/F filters in order to measure suspended sediment concentrations. The filters were dried for 4 days at 60°C before re-weighing. The sediment concentrations used for the calibration ranged from 0 to 2 g/L and were determined from a range of field reference concentrations measured from water samples collected at various depths across the Ameland Inlet during a range of meteorological conditions. The calibration showed very strong correlation between total SSC and NTU (R=1.00), which gives high confidence of the accuracy of the total SSC derived from the OBS measurements (Fig. S3).


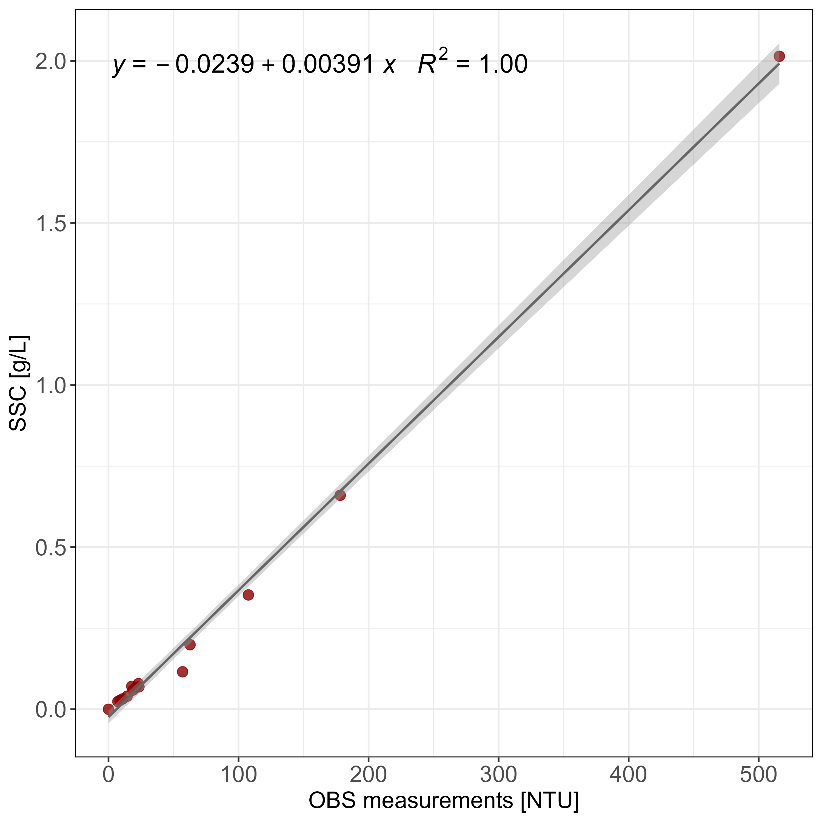
**Figure S3**. Suspended Sediment Concentration (SSC) calibration curve.

The 32 measured SSC profiles are shown in Figure S4, illustrating suspended sediment concentrations over depth. Anomalous values occasionally appeared at the top and bottom of the profiles, typically caused by sensor disturbance: either from contact with the seafloor, which can resuspend additional sediment, or from interference at the water surface during insertion. To correct for this, we removed the measurement immediately following the maximum depth reached during each profile (i.e. the values recorded while retrieving the instrument). This simple correction effectively removed retrieval-related artefacts without requiring a more elaborate filtering approach.


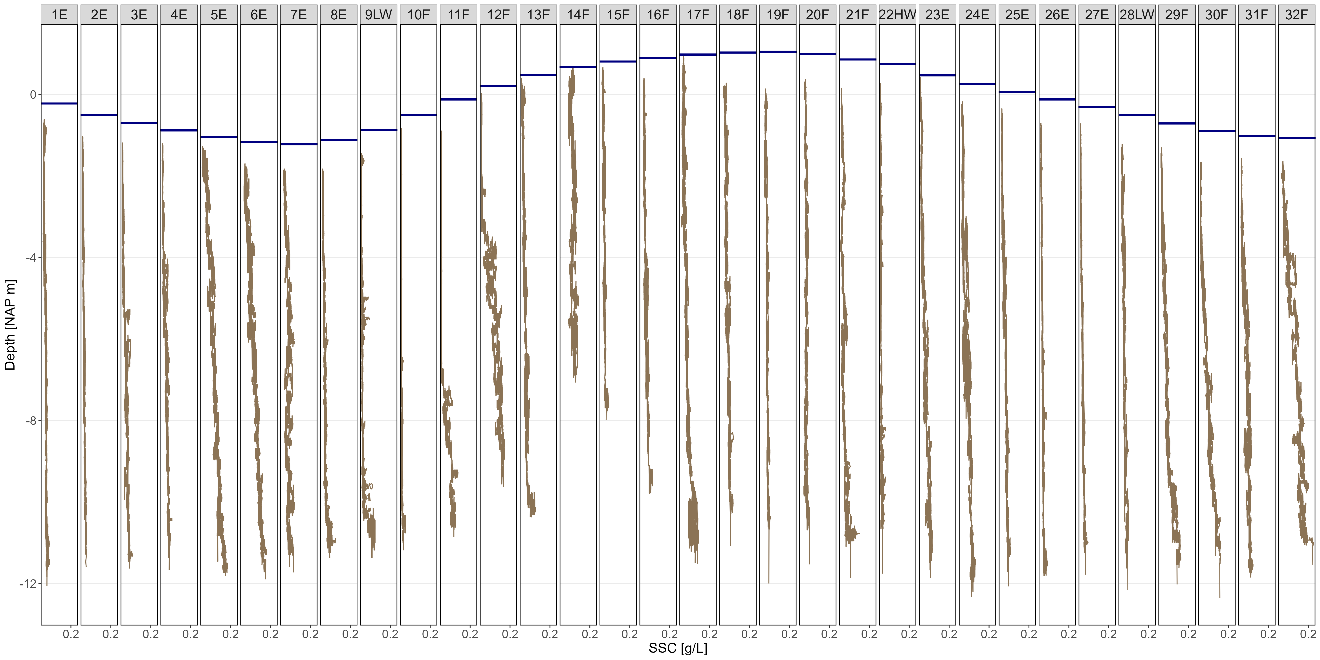


Figure S4. Suspended Sediment Concentration (SSC) profiles.

References

1 Wallinga, J., Murray, A. & Duller, G. Underestimation of equivalent dose in single-aliquot optical dating of feldspars caused by preheating. *Radiation measurements* **32**, 691-695 (2000).

2 Wintle, A. Anomalous fading of thermo-luminescence in mineral samples. *Nature* **245**, 143-144 (1973).

3 Wallinga, J. Optically stimulated luminescence dating of fluvial deposits: a review. *Boreas* **31**, 303-322 (2002). <https://doi.org:https://doi.org/10.1111/j.1502-3885.2002.tb01076.x>

4 Galbraith, R. F., Roberts, R. G., Laslett, G. M., Yoshida, H. & Olley, J. M. Optical dating of single and multiple grains of quartz from Jinmium rock shelter, northern Australia: Part I, experimental design and statistical models. *Archaeometry* **41**, 339-364 (1999).

5 de Boer, A., Kook, M. & Wallinga, J. Testing the performance of an EMCCD camera in measuring single-grain feldspar (thermo) luminescence in comparison to a laser-based single-grain system. *Radiation Measurements* **175**, 107168 (2024). <https://doi.org:https://doi.org/10.1016/j.radmeas.2024.107168>

6 Kook, M., Lapp, T., Murray, A., Thomsen, K. J. & Jain, M. A luminescence imaging system for the routine measurement of single-grain OSL dose distributions. *Radiation Measurements* **81**, 171-177 (2015).

7 Hütt, G., Jaek, I. & Tchonka, J. Optical dating: K-feldspars optical response stimulation spectra. *Quaternary Science Reviews* **7**, 381-385 (1988).

8 Murray, A. *et al.* Optically stimulated luminescence dating using quartz. *Nature Reviews Methods Primers* **1**, 72 (2021). <https://doi.org:10.1038/s43586-021-00068-5>

9 Thomsen, K. J., Murray, A. S., Jain, M. & Bøtter-Jensen, L. Laboratory fading rates of various luminescence signals from feldspar-rich sediment extracts. *Radiation measurements* **43**, 1474-1486 (2008).

10 Murray, A. S. & Wintle, A. G. The single aliquot regenerative dose protocol: potential for improvements in reliability. *Radiation measurements* **37**, 377-381 (2003).

11 Aitken, M. Thermoluminescence dating: past progress and future trends. *Nuclear Tracks and Radiation Measurements (1982)* **10**, 3-6 (1985).

12 Waajen, I. M. *et al.* Late MIS5a in the southern North Sea: new chronostratigraphic insights from the Brown Bank Formation. *Journal of Quaternary Science* **39**, 408-420 (2024).

13 de Boer, A., Seebregts, M., Wallinga, J. & Chamberlain, E. A one-day experiment quantifying subaqueous bleaching of K-feldspar luminescence signals in the Wadden Sea, the Netherlands. *Netherlands Journal of Geosciences* **103**, e22 (2024).

14 Kars, R., Reimann, T. & Wallinga, J. Are feldspar SAR protocols appropriate for post-IR IRSL dating? *Quaternary Geochronology* **22**, 126-136 (2014).

15 de Boer, A., Steinbuch, L., Heuvelink, G. & Wallinga, J. A novel method to assess crosstalk in single-grain luminescence detection. *Radiation Measurements*, 107459 (2025).

16 Kreutzer, M. S. & Developers, C. P. Package ‘Luminescence’. (2025).

17 Spooner, N. A. The anomalous fading of infrared-stimulated luminescence from feldspars. *Radiation Measurements* **23**, 625-632 (1994). <https://doi.org:https://doi.org/10.1016/1350-4487(94)90111-2>

18 Spooner, N. A. On the optical dating signal from quartz. *Radiation Measurements* **23**, 593-600 (1994). <https://doi.org:doi:10.1016/1350-4487(94)90105-8>

19 Colosimo, I. *et al.* The impact of wind on flow and sediment transport over intertidal flats. *Journal of Marine Science and Engineering* **8**, 910 (2020).
